# Hollow Palladium Nanoparticles Facilitated Biodegradation of an Azo Dye by Electrically Active Biofilms

**DOI:** 10.3390/ma9080653

**Published:** 2016-08-04

**Authors:** Shafeer Kalathil, Rajib Ghosh Chaudhuri

**Affiliations:** 1 Division of Biological and Environmental Science & Engineering, King Abdullah University of Science and Technology, Thuwal 23955-6900, Saudi Arabia; 2 Department of Chemical Engineering, Birla Institute of Technology & Science, Pilani-Dubai Campus, Dubai International Academic City, P.O. Box No. 345055, Dubai, UAE; gcrajib@gmail.com

**Keywords:** electrically active biofilm, dye degradation, hollow nanoparticles, catalysis

## Abstract

Dye wastewater severely threatens the environment due to its hazardous and toxic effects. Although many methods are available to degrade dyes, most of them are far from satisfactory. The proposed research provides a green and sustainable approach to degrade an azo dye, methyl orange, by electrically active biofilms (EABs) in the presence of solid and hollow palladium (Pd) nanoparticles. The EABs acted as the electron generator while nanoparticles functioned as the electron carrier agents to enhance degradation rate of the dye by breaking the kinetic barrier. The hollow Pd nanoparticles showed better performance than the solid Pd nanoparticles on the dye degradation, possibly due to high specific surface area and cage effect. The hollow cavities provided by the nanoparticles acted as the reaction centers for the dye degradation.

## 1. Introduction

Azo dyes are commonly used in textile industries and the dyes present in textile wastewater severely threaten aquatic life due to their acute toxicity [1,2]. They bio-accumulate in the environment and cause carcinogenic and mutagenic effects in humans [3]. In addition, dyes are recalcitrant molecules, which make it difficult to remove them from wastewater by conventional wastewater treatment processes [3]. Even though many methods, including chemical and biological methods, are available to degrade dyes, most of them are far from satisfactory [1,2,4,5]. In chemical dye degradation, NaBH_4_ is used as the reducing agent, which causes secondary pollution due to its extreme toxicity [6]. To overcome this chemical toxicity, photodegradation technique is another widely used method for the removal of dyes by using appropriate photocatalyst, either under UV or visible light [7,8]. For this purpose, different kinds of inorganic- or organic-based semiconductors are used by changing parameters like dye or catalyst concentration [9,10,11] for wide varieties of dyes. Major disadvantages of this method are instability of the photocatalysts and incomplete or partial degradation of the dyes [12]. Dye degradation by physical methods such as ultrasound treatments consume high energy and need expensive instrumental set-up [13]. Biodegradation by fungi, bacteria and enzymes are also largely employed to remove dyes from wastewater [5], but the major drawbacks of these biological method are the inherent slow degradation nature and formation of sludge [14]. Electrically active microbes such as *Shewanella oneidensis* MR-1 can effectively degrade azo dyes extracellularly with the aid of outer-membrane c-type cytochromes (OM *c*-Cyts) [15]. Electrically active biofilms (EABs) are commonly used in bioelectrochemical systems (BESs) such as microbial fuel cells (MFCs) as living biocatalysts for electricity generation through the substrate oxidation [2,16,17]. In addition, EABs were employed in synthesis of metal nanoparticles and band gap engineering of semiconductors [18,19,20]. Hollow metal nanoparticles are considered outstanding electron transfer catalysts and are largely employed in various catalytic reactions [21,22,23,24,25]. For example, hollow metal nanoparticles exhibit better catalytic activities on oxygen reduction and methanol oxidation than the solid metal nanoparticles [23]. Mahmoud et al. [23] demonstrated that hollow Pd nanoparticles enhanced 4-nitrophenol reduction by NaBH_4_ over solid Pd nanoparticles. The hollow cavities provided by the nanoparticles acted as the reaction centers due to the cage effect [21,22,23,26]. Here, we employed EABs to degrade methyl orange with hollow Pd nanoparticles as electron transfer agents. The proposed method can be an efficient and green degradation route for the removal of dyes from wastewater through EABs/hollow Pd hybrid system. 

## 2. Results and Discussion

### 2.1. Evaluation of as-Synthesized Pd Nanoparticles

**Figure 1:**
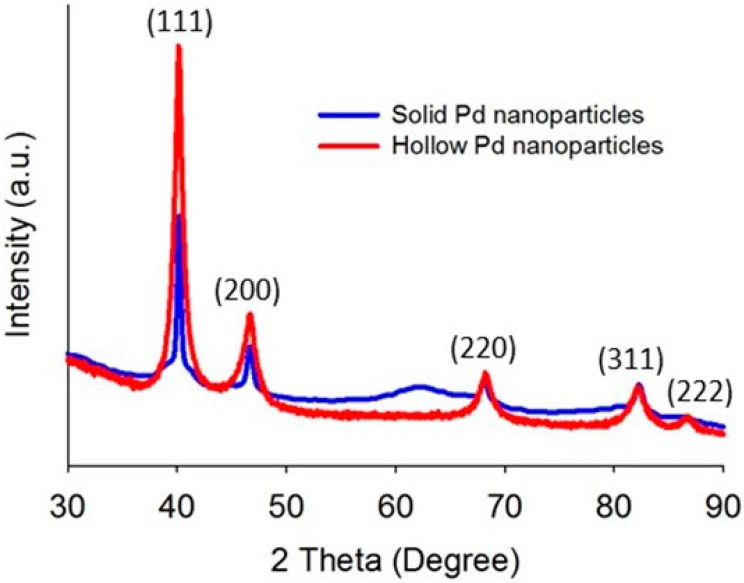
XRD patterns of solid and hollow Pd nanoparticles.

Prior to the application of solid and hollow Pd nanoparticles for the dye degradation, the particles were characterized by X-ray diffraction (XRD) and transmission electron microscope (TEM) to analyze shape, phase and size of the as-synthesized particles. The XRD patterns of solid and hollow Pd are shown in Figure 1. The positions and intensities of the diffraction peaks of hollow (40.16°, 46.67°, 68.23°, 82.16° and 86.75°) and solid Pd nanoparticles (40.15°, 46.64°, 68.07° and 82.21°) are in good agreement with the literature values for cubic Pd (46-1043 from JCPDS PDF Number, Note: (111) plane lattice spacing for cubic Pd is 0.225 nm and space group Fm3m and space group number 225). Although the lattice d-spacing of both solid and hollow nanoparticles are the same (0.225 nm), the crystalline sizes (measured by Scherrer equation) [27,28] of solid and hollow Pd nanoparticles are 9.5 and 12.7 nm, respectively.

**Figure 2:**
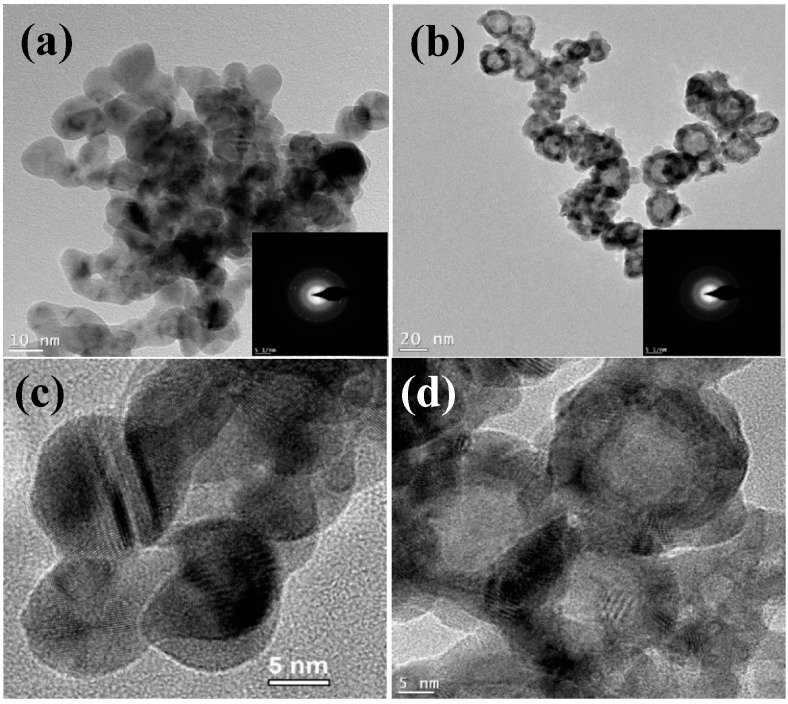
Low and high magnification TEM images of solid (**a**,**c**) and hollow (**b**,**d**) Pd nanoparticles. Insets (**a**,**b**) show selected area electron diffraction (SAED) patterns.

Figure 2 shows the TEM images of solid and hollow Pd nanoparticles with different magnifications and the images clearly depict that both particles are monodispersed with spherical shape. Figure 2a,c and Figure S1 (Supplementary Materials) represent the low and high resolution TEM images of solid Pd nanoparticles. The inset in Figure 2a shows the selected area electron diffraction (SAED) pattern of the solid particles, which clearly shows the polycrystallinity of the nanoparticles. The polycrystallinity was also confirmed from the high resolution TEM image that shows crystalline planes with different d-spacings (0.225, 0.196, 0.1737, and 0.1378 nm) as shown in Figure 2c. The average particles size of solid Pd was calculated as 12.5 nm from the TEM image using image processing software. Figure 2b,d and Figure S2 (Supplementary Materials) represent the low and high resolution TEM images of hollow Pd nanoparticles. The average particles size and shell thickness of the hollow nanoparticles were ~25 and ~8 nm, respectively, measured by the image processing software. The SAED pattern (inset in Figure 2b) showed polycrystalline nature of the hollow Pd nanoparticles.

**Figure 3:**
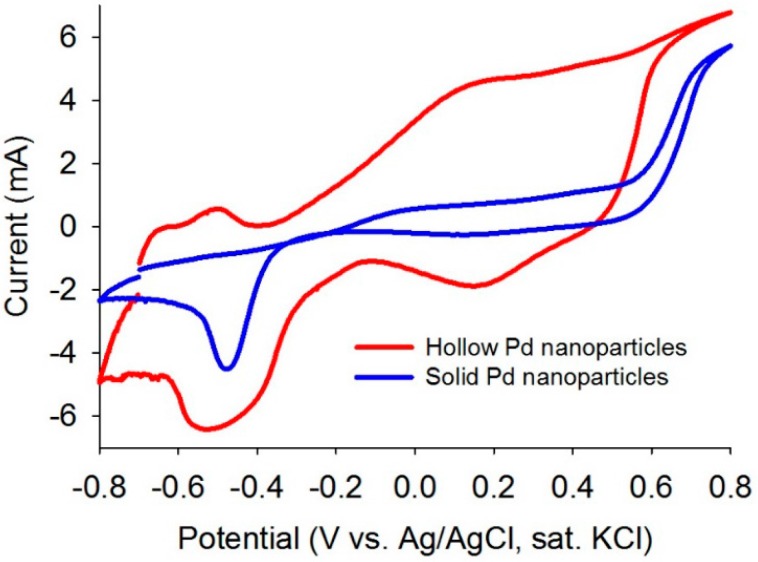
CV plots of the as-synthesized solid and hollow Pd nanoparticles in aqueous KOH (1M) with a scan rate of 20 mV/s (sat: saturated).

Cyclic voltammetry (CV) analyses were performed to evaluate electrocatalytic properties of the as-synthesized solid and hollow Pd nanoparticles. The CV data showed superior electrocatalytic behavior of the hollow Pd compared to the solid Pd by showing excellent redox behavior (Figure 3). This observation indicates that the hollow Pd particles are more active to participate in electrocatalytic reactions than the solid particles.

### 2.2. Evaluation of as-Developed EABs

**Figure 4:**
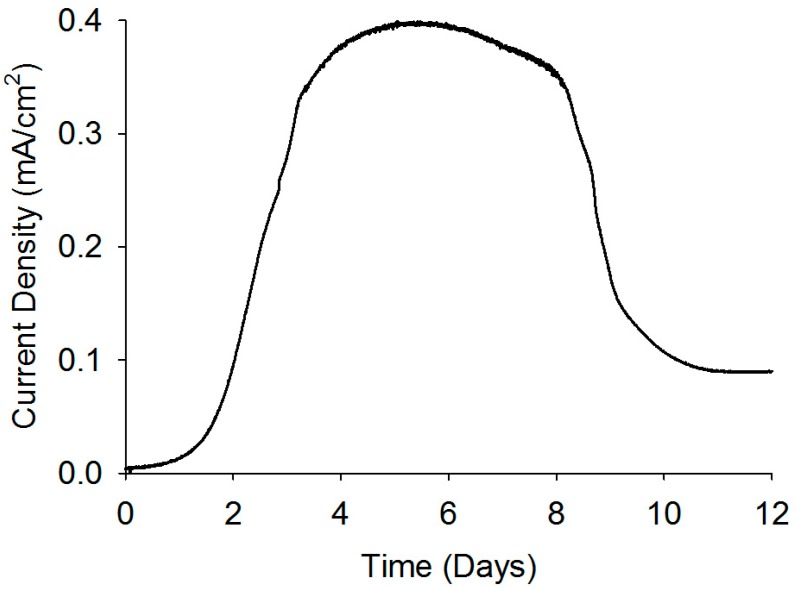
Current generation by the EAB through the substrate oxidation.

**Figure 5:**
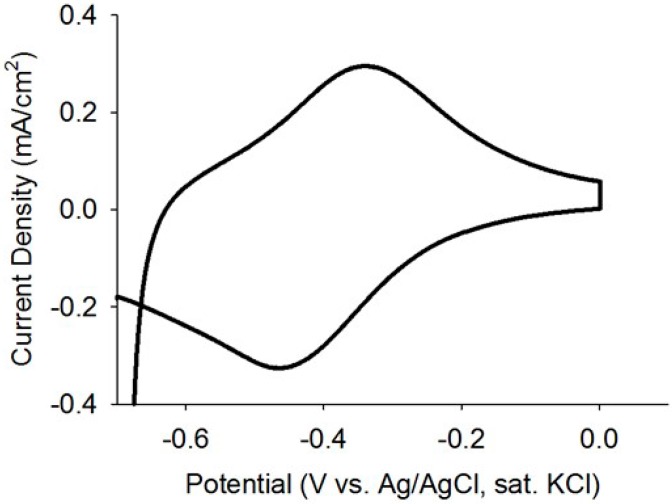
CV plot of the as-developed EABs with a scan rate of 1 mV/s.

Current analysis and CV are considered as powerful tools to investigate the electrochemical activities of biofilms as they can reveal catalytic activities of the EABs [29]. Thus, prior to studying the application EABs for dye degradation, the electrochemical properties of the EABs were analyzed without any catalyst nanoparticles. A gradual increase in the current density was observed after poising a potential and a stable current density of 0.4 mA/cm^2^ was reached after one week of continuous BES operation (as shown in Figure 4). The current generation is comparable with reported values in BESs [30] and this indicated the well-developed EABs formation on the carbon paper. The CV analysis confirmed the electrochemical activity of as-developed EABs on the carbon paper with an oxidation current (Figure 5). As potential raised above a threshold (around −0.6 V) value, the positive anodic current reflected continuous oxidation of acetate. The peak observed at around ~0.360 V (vs. Ag/AgCl) may be originated from the OM *c*-Cyts on the EAB (Figure 5) and the CV plot is similar to previous observations found for *Geobacter sulfurreducen*, the highest electricity producing bacterium till known [29]. Moreover, the appearance of anodic and cathodic peaks may be attributed to pseudo capacitance behavior of OM *c*-Cyts [31]. As-developed EABs were employed for the MO degradation in presence of hollow or solid Pd nanoparticles. 

### 2.3. Biodegradation of MO

The EABs oxidized the acetate by generating electrons and thoseelectrons reduced the methyl orange (MO). The EABs acted as the electron generator and the MO as the electron acceptor. The dye degradation by the EABs is thermodynamically favorable, but it is kinetically very slow in the absence of any catalysts [32]. The Pd nanoparticles helped to overcome the kinetic barrier by providing an electron transfer channel between the EABs and dye molecules. The rate of MO degradation by the EABs was dependent on the presence of these Pd nanoparticles. 

**Figure 6:**
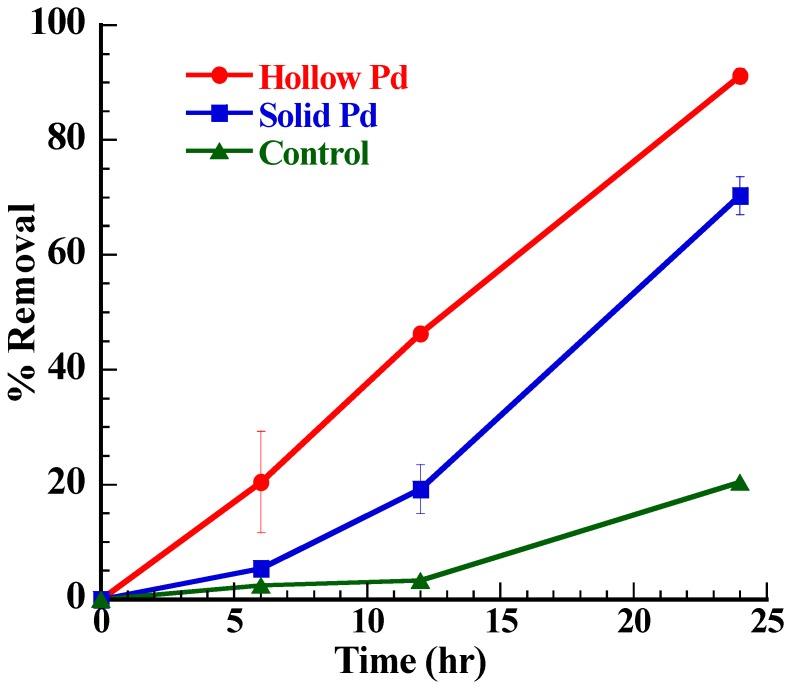
Plot of percent removal of MO dye in the solution with time by EABs in presence of solid or hollow Pd nanoparticles.

The EABs with hollow Pd nanoparticles exhibited enhanced MO degradation as compared to the EABs with solid Pd nanoparticles and EABs (control) only (Figure 6). As shown in Figure 6, in certain time limit (24 h), the order of percent removal of dye is hollow particles > solid particles > without particles (control). The maximum percent removal of dye was observed in the case of hollow particles (~95% in 30 h), followed by solid particles (~85% in 48 h) and without catalyst (~61% in 72 h). The major reason behind this observation is amount active surface area that takes part has an important role in the electron carrying process. In the case of solid Pd nanoparticles, only surface atoms were actively participating in the reaction while the hollow Pd nanoparticles provided surface atoms, inner hollow cavities (cage effect) and porous layer for the catalytic reaction [21,22,23]. Moreover, the hollow Pd nanoparticles showed higher BET surface area (426.2 m^2^/g) than the solid Pd nanoparticles (76.74 m^2^/g). The higher surface area of the hollow Pd nanoparticles allowed adsorbing more MO on the surface for the catalytic degradation, whereas the cage structure favored storing excess electrons within the cavities. In addition, the unique hollow structure of Pd nanoparticles, which combines nano and micro scale properties facilitated electron transfer from the EABs to MO and because of that the degradation rate in the presence of hollow particles (0.1477 h^−1^) was higher than that of the solid Pd nanoparticles (0.0666 h^−1^). The Pd nanoparticle possesses higher conductivity than the bacterial nanowire which has been generally believed to be an electrical channel for the bacterial EET [33]. In addition, Pd nanoparticles show high catalytic activities for various redox reactions [34]. The CV analysis confirmed the outstanding redox behavior of the hollow Pd nanoparticles over the solid Pd nanoparticles (Figure 3). Because of these properties, hollow Pd nanoparticles significantly enhanced the bacterial EET process by binding with outer membrane proteins [35] and the enhanced bacterial EET accelerated the MO degradation. 

### 2.4. Possible Dye Degradation Mechanism

**Scheme 1:**
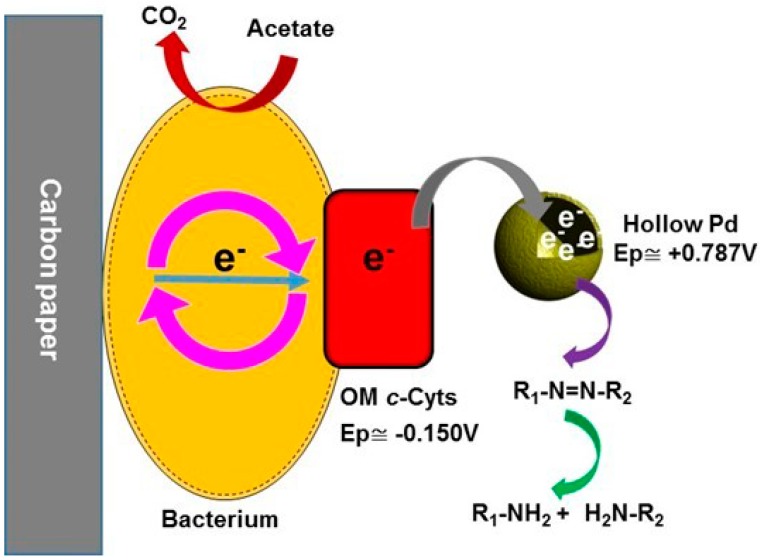
Proposed mechanism of the dye degradation by electrically active biofilms (EABs) in the presence of hollow Pd nanocatalysts.

Bacterial decolorization of dye is considered an extracellular process because of its large size and polarity and OM *c*-Cyts are believed to be the main contributors for the extracellular dye degradation [15]. The reduction potential of OM *c*-Cyts is −0.150 V (vs. Ag/AgCl) [36] while for Pd^2+^/Pd metal is +0.787 (vs. Ag/AgCl) [34]. Here, the reduction potential of the Pd is more positive than the OM *c*-Cyts, which allowed fluent electron transfer from the OM *c*-Cyts to the Pd nanoparticles. Eventually, the electrons captured by the Pd nanoparticles reduced the adsorbed dye on the surface of the nanoparticles (Scheme 1). There was no dye degradation in the absence of either EABs or acetate, indicating the inevitable roles of EABs and acetate in the MO degradation (Figure S3). Addition of a biocide (1% glutarldehyde) largely suppressed the MO degradation, which revealed that live biofilm was responsible for the dye degradation (Figure S3). In addition, this observation further confirmed that the dye removals through adsorption by the catalysts and biofilm were negligible. 

## 3. Experimental Section

### 3.1. Material

All chemical reagents used for these experiments were taken from following companies: CoCl_2_∙6H_2_O (Sigma-Aldrich, St. Louis, MO, USA, 99%–102% assay), citric acid (Fisher, Hampton, NH, USA), NaBH_4_ (Fluka, ≥99%), PdCl_2_ (Acros 59% Pd), methyl orange (Acros, Geel, Belgium), and sodium acetate (Sigma-Aldrich, ≥99%). All chemicals were of analytical grade and used as received. Deionized Milli-Q water was used as solvent for all chemical reaction.

### 3.2. Catalyst Synthesis

Hollow Pd nanoparticles were synthesized by Kirkendall method using Co as sacrificial template [28,37]. Initially, Co nanoparticles were synthesized at anaerobic condition by the reduction of CoCl_2_ with NaBH_4_ in aqueous citric acid media, then PdCl_2_ solution was added to the Co solution at anaerobic condition to form Pd nanoparticles through auto-oxidation of Co nanoparticles because of its low reduction potential (Co^2+^/Co, −2.8 V vs. SHE, standard hydrogen electrode) than Pd^2+^/Pd couple (0.59 V vs. SHE). The hollow structured Pd was formed (Scheme S1 in Supplementary Materials) because of the sacrificial nature of Co nanoparticle and higher diffusion rate of Co^2+^compared to Pd because of low atomic mass of Co. The chemical reaction of Pd formation can be written as $Co\;(S)+Pd^{2+}\to Pd\;(S)+Co^{2+}$.

Solid Pd nanoparticles were synthesized by same reaction condition without Co nanoparticles to compare the enhanced catalytic activity of the hollow structures.

### 3.3. Electrically Active Biofilm Formation

Electrically active biofilm (EAB) was developed using a three-electrode bioelectrochemical system (BES). Three-electrode BES system is a conventional way to develop EABs on various electrode surfaces [36]. A plain carbon paper (2 × 4 cm^2^), Pt mesh and Ag/AgCl (Sat. KCl) were used as working, counter and reference electrodes, respectively. Artificial wastewater medium (95 mL) [17] was used as the medium and the medium was purged with N_2_ for 30 min to make the medium strictly anaerobic. Five milliliters of anaerobic sludge (collected from KAUST wastewater treatment plant) was added to the medium as the bacterial inoculum with acetate (10 mM) as the electron donor. The use of mixed culture consortia is attractive to develop EABs as they are readily obtainable in large quantities and are tolerant to environmental stress and fluctuations [38]. We used acetate as the electron donor as it can induce electrically active microbes [39]. A poising potential of –0.2 V (vs. Ag/AgCl) was applied across the working electrode to drive the bacterial metabolism. The medium was changed (no more inoculum) every two days and the system was continuously operated for 1 week until a stable current was observed. 

### 3.4. Characterization of Catalyst

Before experimental study on the degradation of methyl orange, the catalysts as well as the EABs were characterized by several techniques to analyze the actual properties of these materials. X-ray diffraction (XRD) analyses were performed for solid and hollow catalysts using D8 Venture diffractometer instruments (Bruker Corporation, Bremen, Germany) with scanning rate of 0.03°/s in the 2θ range of 20° to 90° in the form of a layer on the glass plate. TEM analyses of solid and hollow Pd nanoparticles were performed with Titan 80–300 instruments (FEI, Eindhoven, The Netherlands). Alcoholic suspension of these catalysts were sonicated for 15 min in bath sonicator and dropped on the carbon coated Cu grid. The surface area was measured by BET analysis with Tristar II Micromeritics instruments (Micromeritics, Norcross, GA, USA). The samples were degassed overnight at 120 °C prior to analysis.

CV measurement of as-synthesized catalysts was conducted using a three-electrode system. A glassy carbon was used as working electrode (WE), Pt mesh as counter electrode and Ag/AgCl (Sat. KCl) as reference electrode. The WE was prepared by coating solid or hollow Pd nanoparticles onto the glassy carbon disk electrodes. Briefly, samples (5 mg) were dispersed in ethanol (3:2) solution and ultrasonicated for 10 min for uniform mixing. Ten microliters of well-dispersed Pd catalysts were applied onto the glassy carbon disk electrode (5 mm in diameter). After drying at room temperature, 3 µL of Nafion (0.05 wt %) was applied onto the surface of the catalyst layer to form a thin protective film. KOH (1M) was used as the electrolyte. CV analysis of the EABs was also performed using the three-electrode system. After obtaininga stable current by the EABs developed on the carbon paper, CV was performed with a scan rate of 1 mV/s at anaerobic condition as previously reported [29]. 

### 3.5. Degradation of Methyl Orange

As-developed EABs on the carbon paper was dipped in the artificial wastewater medium (100 mL) [17] containing 10 mM acetate (electron donor), 5 mM methyl orange (MO) and 20 mg/L catalyst (hollow or solid Pd nanoparticles). Prior to the EABs dipping, the medium was sonicated for 30 min to make homogeneous dispersion of the catalyst and also the medium was purged with N_2_ to make the system anaerobic. During the degradation experiment, the medium was kept under a slow magnetic stirring to prevent mass transfer limitation. A control experiment without catalyst (only EABs) was also performed for comparison. To investigate the adsorption activity of the catalysts, an experiment by adding catalyst without dipping the EAB was conducted. The samples were collected every 6 h using a syringe. All experiments were conducted in triplicate and average values were reported.

## 4. Conclusions

Although dye degradation by EABs is thermodynamically favorable, the degradation is sluggish due to the high kinetic barrier. The addition of metallic nanoparticles significantly enhanced the degradation rate by increasing the electron transfer rate from EABs to dye molecules. Especially, the addition of hollow Pd nanoparticles into the EABs system significantly enhanced (four times) the dye degradation as compared to EABs only. The catalytic activity of hollow nanoparticles was much higher than that of the solid nanoparticles because of the cage effect and large specific surface area of the former. The study proposes a sustainable approach for the efficient dye degradation through EABs/hollow nanoparticle hybrid system.

## Supplementary Materials

The following are available online at www.mdpi.com/1996-1944/9/8/653/s1. Figure S1: High resolution TEM images of solid Pd nanoparticles; Figure S2: High resolution TEM images of hollow Pd nanoparticles; Figure S3: Plot of percent removal of MO dye in the solution with time at different conditions (control experiments); Scheme S1: Schematic of Pd hollow nanoparticles formation through Kirkendall method using Co as sacrificial template.
