# Supplementary material for: Hollow Palladium Nanoparticles Facilitated Biodegradation of an Azo Dye by Electrically Active Biofilms
Source: Materials (Basel). 2016 Aug 4;9(8):653. doi: 10.3390/ma9080653 (PMC5509264; doi:10.3390/ma9080653)
Supplement: Supplementary file 1 [file materials-09-00653-s001.pdf]

Supplementary Materials: Hollow Palladium Nanoparticles Facilitated Biodegradation of an Azo Dye by Electrically Active Biofilms

Shafeer Kalathil and Rajib Ghosh Chaudhuri

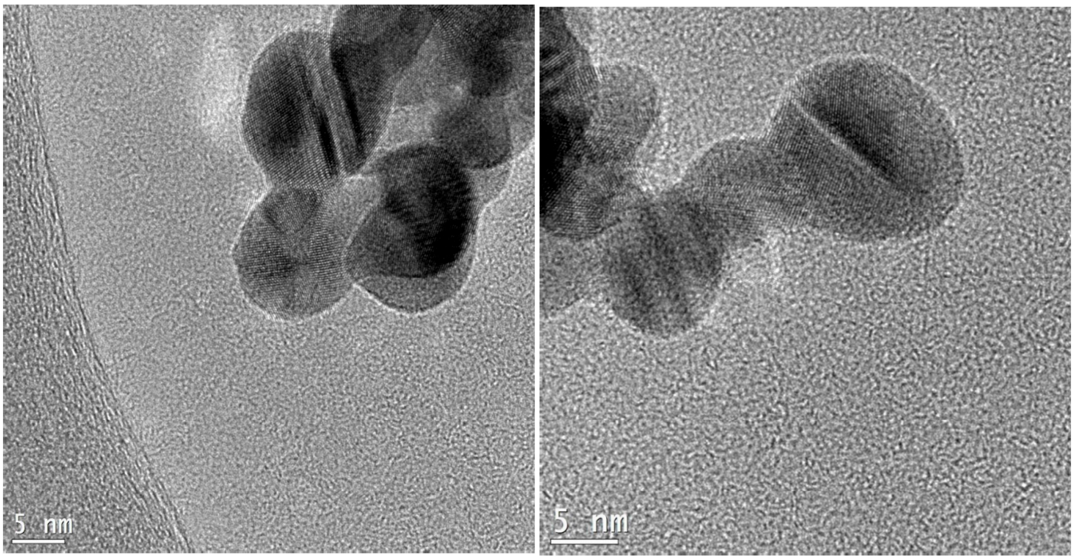

Figure S1. High resolution TEM images of solid Pd nanoparticles.

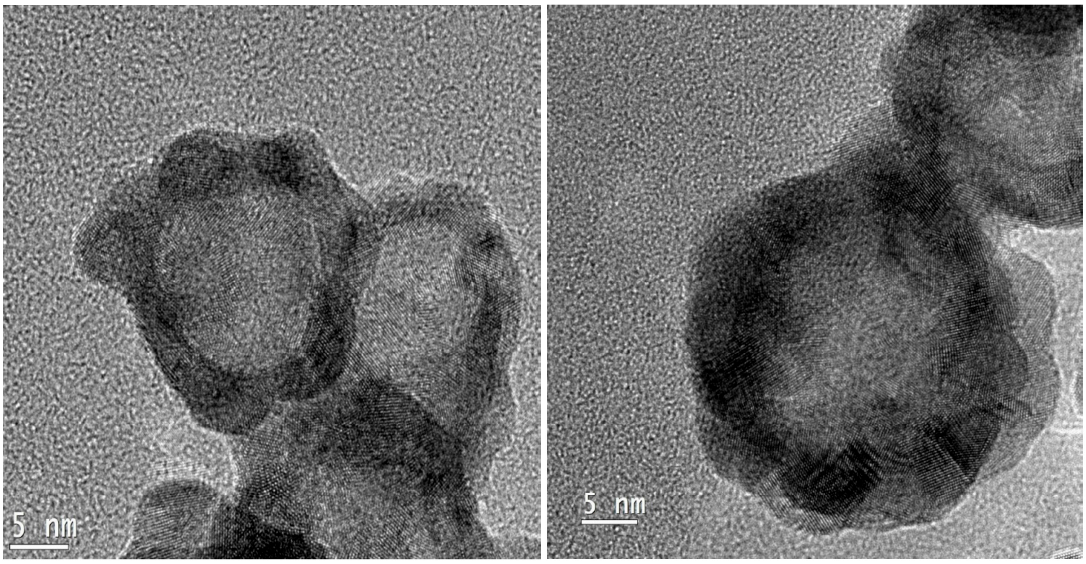

Figure S2. High resolution TEM images of hollow Pd nanoparticles.

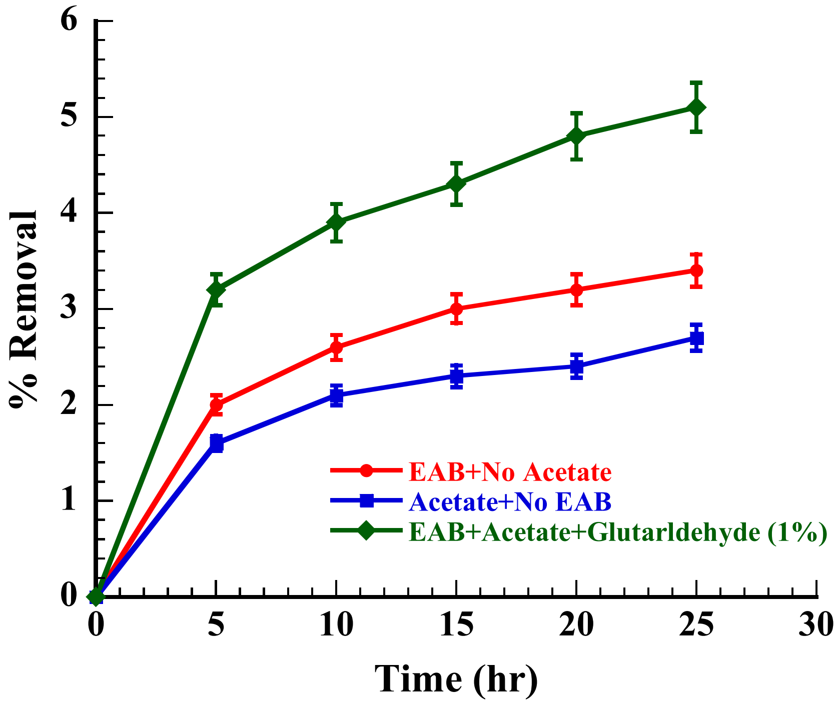

Figure S3. Plot of % removal of MO dye in the solution with time at different conditions (control experiments).

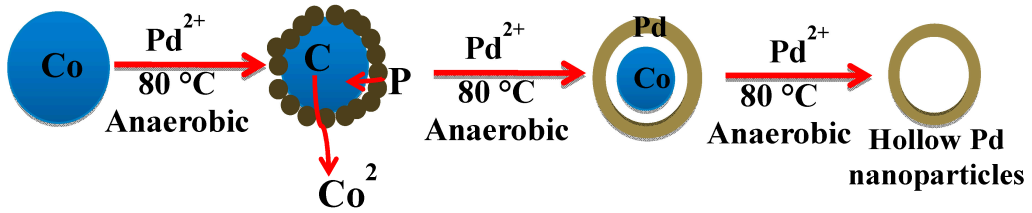

Scheme S1. Schematic of Pd hollow nanoparticles formation through Kirkendall method using Co as sacrificial template.
